# Nucleus Accumbens MC4-R Stimulation Reduces Food and Ethanol Intake in Adult Rats Regardless of Binge-Like Ethanol Exposure during Adolescence

**DOI:** 10.3389/fnbeh.2017.00167

**Published:** 2017-09-07

**Authors:** Francisca Carvajal, José M. Lerma-Cabrera, Manuel Alcaraz-Iborra, Montserrat Navarro, Todd E. Thiele, Inmaculada Cubero

**Affiliations:** ^1^Institute of Biomedical Sciences, Universidad Autonoma de Chile Santiago, Chile; ^2^Department of Psychology, University of Oviedo Oviedo, Spain; ^3^Department of Psychology, University of Almeria Almeria, Spain; ^4^Department of Psychology and Neuroscience, University of North Carolina Chapel Hill, NC, United States

**Keywords:** melanocortin, MC4-R, binge-like ethanol exposure, adolescence, intermittent-access ethanol paradigm, nucleus accumbens

## Abstract

The melanocortin (MC) system regulates feeding and ethanol consumption. Recent evidence shows that melanocortin 4 receptor (MC4-R) stimulation within the nucleus accumbens (NAc) elicits anorectic responses and reduces ethanol consumption and ethanol palatability in adult rats. Ethanol exposure during adolescence causes long-lasting changes in neural pathways critically involved in neurobehavioral responses to ethanol. In this regard, binge-like ethanol exposure during adolescence reduces basal alpha-melanocyte-stimulating hormone (α-MSH) and alters the levels of agouti-related peptide (AgRP) in hypothalamic and limbic areas. Given the protective role of MC against excessive ethanol consumption, disturbances in the MC system induced by binge-like ethanol exposure during adolescence might contribute to excessive ethanol consumption during adulthood. In the present study, we evaluated whether binge-like ethanol exposure during adolescence leads to elevated ethanol intake and/or eating disturbance during adulthood. Toward that aim, Sprague-Dawley rats were treated with ethanol (3 g/kg i.p.; BEP group) or saline (SP group) for 14 days (PND 25 to PND 38). On PND73, all the groups were given access to 20% ethanol on an intermittent schedule. Our results showed that adult rats given intermittent access (IAE) to 20% ethanol achieved high spontaneous ethanol intake that was not significantly enhanced by binge-like ethanol pretreatment during adolescence. However, BEP group exhibited an increase in food intake without a parallel increase in body weight (BW) relative to SP group suggesting caloric efficiency disturbance. Additionally, we evaluated whether binge-like ethanol exposure during adolescence alters the expected reduction in feeding and ethanol consumption following NAc shell administration of a selective MC4-R agonist in adult rats showing high rates of ethanol consumption. For that, animals in each pretreatment condition (SP and BEP) were divided into three subgroups and given bilateral NAc infusions of the selective MC4-R agonist cyclo(NH-CH_2_-CH_2_-CO-His-D-Phe-Arg-Trp-Glu)-NH_2_ (0, 0.75 or 1.5 μg). Results revealed that MC4-R stimulation within the NAc reduced feeding and ethanol intake in high ethanol-drinking adult rats, regardless of previous binge-like ethanol exposure during adolescence, which adds new evidence regarding the dual ability of MC compounds to control excessive ethanol and food intake.

## Introduction

The melanocortin (MC) system is an important neural system involved in the regulation of energy balance and feeding behavior (for review see Gantz and Fong, 2003). Alpha-melanocyte-stimulating hormone (α-MSH) is an endogenous agonist of this system, and agouti-related peptide (AgRP) an inverse agonist that exerts the opposite effect on the same MC receptors (Cone, 2005; De Jonghe et al., 2011). The role of MC signaling within the hypothalamus in modulating feeding behavior, body weight (BW) regulation (Adan et al., 2006; Krashes et al., 2016; You et al., 2016) and food selection (Hillebrand et al., 2006) by homeostatic mechanisms (Pandit et al., 2011; Lerma-Cabrera et al., 2012) has been well established. Moreover, the MC system, especially the melanocortin 4 receptor (MC4-R), are currently a promising target system for the development of drugs intended to treat obesity and eating disorders in humans (Hillebrand et al., 2006; Moriya et al., 2006; Laviano et al., 2008; Steinman and DeBoer, 2013; Girardet and Butler, 2014).

Pharmacological and genetic studies have provided additional evidence that MC plays an important role in modulating neurobiological responses to ethanol, indicating that MC compounds may hold potential for treating alcohol abuse disorders. For example, intracerebroventricular (i.c.v.) infusion of the nonselective MC3/4-R agonist, melanotan-II (MTII) significantly reduces ethanol consumption in alcohol-preferring AA rats (Ploj et al., 2002) and C57BL/6J mice (Navarro et al., 2003). Conversely, i.c.v. infusion of AgRP-(83–132), a nonselective MC3/4-R inverse agonist, increases voluntary ethanol consumption in C57BL/6J mice (Navarro et al., 2005). Consistent with pharmacological studies, genetic deletion of AgRP blunts ethanol self-administration and binge-like ethanol drinking (Navarro et al., 2009). Furthermore, it was found that i.c.v. infusion of MTII reduces ethanol intake in mutant mice lacking MC3-R (MC3R−/−; Navarro et al., 2005) but failed to alter ethanol drinking in MC4R−/− mice (Navarro et al., 2011), suggesting that the protective effects of MC are modulated by MC4-R signaling. We have recently provided additional evidence that MC4-R stimulation within the nucleus accumbens (NAc) elicits anorectic responses and reduces ethanol consumption in rats that drink ethanol at moderate rates (Lerma-Cabrera et al., 2012), and the action of MC on MC4-R in the NAc helps modulate non-homeostatic aspects (palatability) of ethanol consumption (Lerma-Cabrera et al., 2013b). However, whether MC4-R signaling within the NAc plays a role in ethanol intake and/or feeding in rats showing high rates of ethanol intake remains unexplored.

Binge-drinking, defined as drinking excessive amounts of alcohol in a short period of time, is a popular pattern of consumption within the adolescent population (Pedersen and von Soest, 2015; Tanumihardjo et al., 2015). Adolescence is an important period of brain development during which heavy drinking triggers long-lasting adaptive changes in neural pathways critically involved in neurobehavioral responses to ethanol (Pascual et al., 2007, 2009, 2012; Maldonado-Devincci et al., 2010b). Several studies have shown that early alcohol use correlates with an increased likelihood of alcohol abuse (Pascual et al., 2009; Maldonado-Devincci et al., 2010a) and eating disorders in adulthood (Stickley et al., 2015). However, the underlying mechanisms leading to enhanced vulnerability to alcohol abuse and eating disorders after ethanol exposure during adolescence are still unclear.

Importantly, we have demonstrated that binge-like ethanol exposure during adolescence reduces basal α-MSH activity in hypothalamic and limbic areas and alters AgRP responses to acute ethanol administration (Lerma-Cabrera et al., 2013a). These data, together with previous evidence showing a key role of MC in feeding and ethanol drinking, suggest that changes in the MC system induced by binge-like ethanol exposure during adolescence may contribute to excessive ethanol consumption and/or eating disturbances during adulthood.

First, the present study assessed whether binge-like ethanol exposure during adolescence leads to elevated ethanol intake in adult rats with high rates of ethanol consumption as a consequence of exposure to an intermittent-access (IAE) 20% ethanol-drinking paradigm. Additionally, we evaluated whether binge-like ethanol exposure during adolescence alters the expected reduction in feeding and ethanol consumption following NAc shell administration of a selective MC4-R agonist in adult rats showing high rates of ethanol consumption.

## Materials and Methods

### Animals

Male Sprague-Dawley rat pups on postnatal day (PND 21; Charles River Laboratories, Spain) arrived at the laboratory and were housed in groups of four rats per cage in an environmentally controlled room (22°C temperature on a 12:12 h light-dark cycle with lights off at 8 am). After an acclimatization period of 4 days (until PND 25) during which the animals were handled briefly every day, the paradigm of binge-like ethanol exposure began. At adulthood, rats were cannulated bilaterally in the NAc and thereafter were housed individually. Standard rodent chow and water were provided *ad libitum* throughout the experiments, and the manipulations were conducted at the onset of the dark phase. All the procedures used were in accordance with the animal care guidelines established by Spanish Royal Decree 53/2013 and were approved by the University of Almeria Bioethical Animal Care and Use Committee.

#### Ethanol Exposure

**Figure 1:**
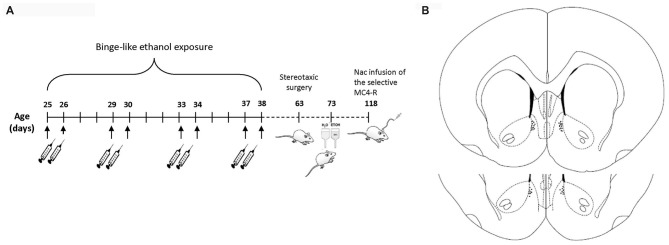
**(A)** Illustration of the timeline of binge-like ethanol exposure, behavioral measures and pharmacological treatment. **(B)** Histological reconstruction of location of injection sites in the Nucleus accumbens (NAc) adapted from Paxinos and Watson (1998).

Morning doses of either 25% (w/v) ethanol (3.0 g/kg) in isotonic saline (binge ethanol pretreatment group, BEP) or saline (saline pretreatment group, SP) were administered intraperitoneally (i.p.) to 25-day-old pups on a 4-day cycle, consisting of two injection days followed by 2 days without injections, for 2 weeks. Specifically, pups were injected at PND 25, 26, 29, 30, 33, 34, 37 and 38 (Pascual et al., 2009; Lerma-Cabrera et al., 2013a). Consistent with previous studies, this paradigm of binge-like ethanol exposure produced a blood ethanol concentration (BEC) of 210 ± 11 mg/dl 30 min after each single dose of ethanol (Pascual et al., 2007; Figure 1A).

#### Surgery

Twenty-five days after the last ethanol injection administered during adolescence (adult stage, PND 63), rats were anesthetized with Equithesin (0.3 ml/100 g) for cannula placement. Bilateral 26-G cannulae (Plastics One, Inc., Germany) were aimed at the NAc shell, using the following stereotaxic coordinates (Paxinos and Watson, 1998): AP: −1.7 mm, ML: ±0.8 mm and DV: −5.4 mm. The rats were allowed to recover for 10 days before any experimental manipulation.

During the recovery phase, animals were maintained warm until righting reflex has returned and also, sterile isotonic fluid was administered subcutaneously to facilitate proper hydration. Finally, to minimize wound contamination, a new cage with clean bedding is provided. During the first week after surgery, the general condition of the animal (BW, food/water intake, sign of pain or distress…) was monitored according to a monitoring protocol adapted from Morton and Griffiths (1985). Also, sign of infection at the incision were checked daily and treated when it needed.

When all procedures were complete, cannula placement was histologically verified (Figure 1B). Animals with injector tips outside the target regions (1–2 per treatment in each group) were discarded from the analysis, yielding final group sizes of *n* = 20 for the BEP group and *n* = 24 for the SP group.

### Behavioral Procedures

#### Intermittent-Access 20% Ethanol 2-Bottle-Choice Drinking Paradigm

After 10 days of postsurgical recovery and before NAc infusion of the selective MC4-R agonist, the rats received 24 h of free access to two bottles (one containing water and the other containing 20% (v/v) ethanol) three times per week across 45 days as described previously by Simms et al. (2008). Given that ethanol concentration (25%) used in the protocol of binge-like ethanol exposure is probably to lead to aversive reactions, we decided to use the same ethanol concentration proposed by Simms et al. (2008). On Monday, Wednesday and Friday, each rat was given access to one bottle of 20% (v/v) ethanol and one bottle of water. After 24 h, the ethanol bottle was replaced with a second water bottle that was available for the next 24 h. Thus, the animals were exposed to a total of 19 sessions in which the ethanol bottle was available. The positions of the bottles were changed every ethanol drinking session to control for position preferences. To obtain measures of consumption that corrected for individual differences in rat size, we calculated intake in g of ethanol consumed per kg of BW for each 24-h measurement of ethanol intake (g/kg/24 h). Throughout the experiments, intake measures (ethanol, food and water) and BW were assessed.

#### Procedure for NAc Administration of the Selective MC4-R Agonist

Following 45 days during which baseline intake measures were collected (Figure 1A), both the BEP and SP groups were distributed into three subgroups matched for ethanol consumption (g/kg/24 h) over the final 3 days of IAE. In this way, we ensured that the baseline level of ethanol consumption was equal between the groups. One hour before the beginning of the dark phase of the light:dark cycle, the rats were weighed and the ethanol, water and food were removed from the cages. Then, isotonic saline or the selective MC4-R agonist cyclo(NH-CH_2_-CH_2_-CO-His-D-Phe-Arg-Trp-Glu)-NH_2_ (Phoenix Pharmaceuticals, Inc., Belmont, CA, USA) at one of two possible doses (1.5 or 0.75 μg) was bilaterally injected at a volume of 0.5/site into the NAc (*n* = 10 per treatment in each group). The MC4-R agonist used is 90-fold and 3400-fold selective for MC4-R over MC3-R and MC5-R, respectively (Bednarek et al., 2001). We have previously found that the selected doses of that MC4-R agonist were effective in reducing feeding and ethanol consumption in Sprague-Dawley rats (Lerma-Cabrera et al., 2012). Site-directed infusions were given manually over a 1-min period using a 1.0 μl Hamilton syringe connected to a 33-G injector cannula (Plastics One, Inc., Germany) that extended 1 mm beyond the guide cannula. The injector was left in place for an additional 60 s to prevent reflux. Then, rats were immediately returned to their home cage. Within a period of 15 min after drug treatment, ethanol, water and food were returned to the cages. Previous evidence showed that the highest level of food intake in rats occurs during the first 4-h of dark cycle (Tabarin et al., 2007). Moreover, given that differences in food and ethanol intake could vary over this time as described in Lerma-Cabrera et al. (2012), non-cumulative intake measures were collected in two 2-h intervals following drug administration.

#### Blood Ethanol Concentration (BEC)

One week after NAc administration of the selective MC4-R agonist, when consumption measures had stabilized and returned to baseline levels, approximately 10 μl of blood was collected from the tail vein of each rat 4 h after the beginning of the ethanol exposure session. The samples were centrifuged, and 5 μl of plasma from each sample was analyzed for BEC measured in mg/dl (Analox Instruments, Lunenburg, MA, USA). Additionally, 4-h ethanol consumption was recorded (g/kg/4 h) to study the correlation between the level of ethanol intake and BEC for SP and BEP animals.

### Data Analyses

Data from the intermittent-access ethanol drinking paradigm were analyzed using a repeated measures analysis of variance (ANOVA) with pretreatment (BEP and SP group) as the between-subject variable. Non-cumulative ethanol and food intake data were compared using a two-way (drug × pretreatment) repeated measures ANOVA that evaluated the short-term effects of NAc-administration of two doses of the selective MC4-R agonist or saline, at 2 and 4 h after infusion in binge ethanol- or saline-pretreated rats during adolescence. Additionally, total calories (cal/kg/4 h; calories from chow + ethanol solution) consumed in the first 4 h before treatment were analyzed with 3 × 2 (drug × pretreatment) ANOVAs. Finally, one-way ANOVAs were used to analyze BECs (mg/dl) and ethanol consumption (g/kg/4 h). When significant differences were found (*p* < 0.05), pairwise comparisons were conducted with a *post hoc* Fisher’s LSD test. All data are presented as the means ± SEM.

## Results

### Intermittent-Access 20% Ethanol Drinking Paradigm in Saline (SP) and Binge-Ethanol (BEP) Pretreated Groups: Measures of Ethanol and Food Intake

**Figure 2:**
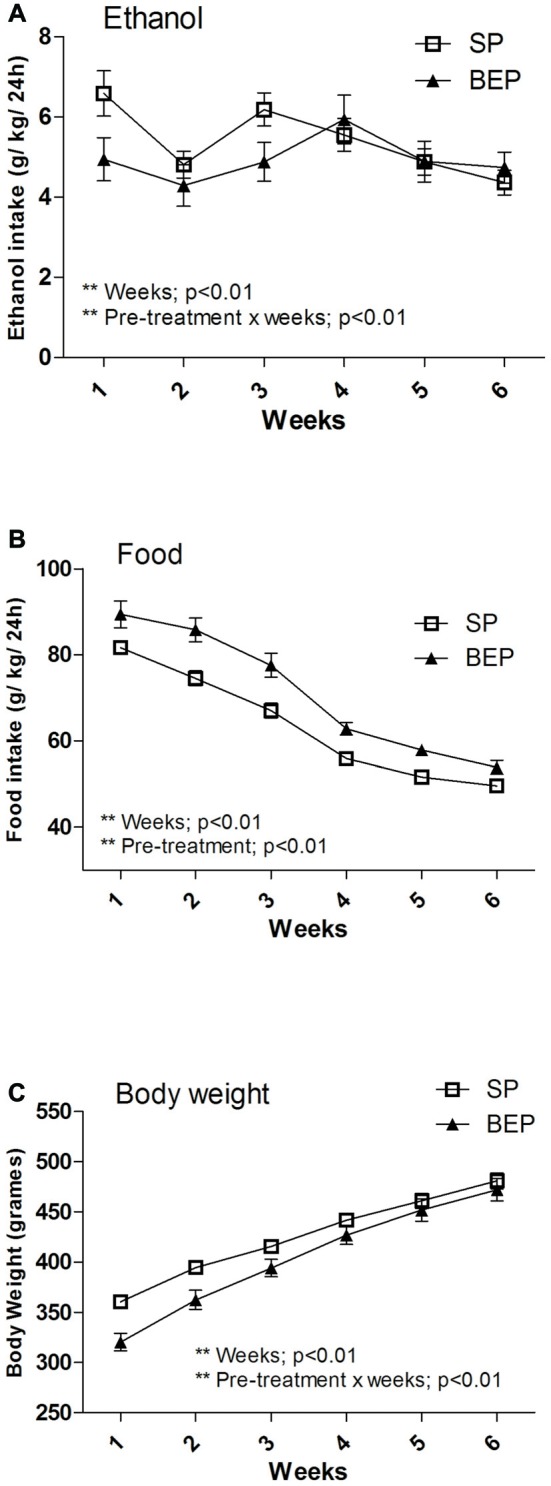
Weekly average 24-h ethanol consumption **(A)**, food intake **(B)** and body weight (BW) **(C)**, collected for the first six complete weeks in an intermittent-access (IAE) 20% ethanol 2-bottle-choice drinking paradigm in both the binge-like ethanol pre-treatment (BEP) and saline pretreatment (SP) groups. Panel **(A)** shows baseline ethanol consumption of 4.36 ± 0.31 and 4.74 ± 0.39 g/kg/24 h for the SP and BEP groups, respectively. All values are means ± SEM.

In agreement with previous reports (Simms et al., 2008; Hwa et al., 2011), the intermittent-access 20% ethanol 2-bottle-choice drinking procedure induced high levels of ethanol intake in both the SP and BEP groups (average ethanol consumption during the last week: SP = 4.36 ± 0.31 g/kg/24 h and BEP = 4.73 ± 0.4 g/kg/24-h). Data showing 24-h voluntary ethanol consumption in the SP and BEP groups using the intermittent-access 20% ethanol drinking paradigm are presented in Figure 2A. A repeated-measures ANOVA performed on ethanol consumption data over 6 weeks revealed a significant main effect of week (*F*_(5,215)_ = 6.00; *p* < 0.01) and a pretreatment × week interaction (*F*_(5,215)_ = 3.48; *p* < 0.01). With respect to the main effect of time, the data revealed that ethanol intake was higher during the first, third and fourth weeks (*p* < 0.01 for all comparisons). Further pairwise comparison using Fisher’s LSD test revealed that the SP group drank significantly more ethanol than the BEP group during the first week of ethanol consumption (*p* < 0.05); however, these differences disappeared over time. At the end of the experimental procedure, the two groups showed similar, high rates of voluntary ethanol consumption.

Data showing 24-h food intake in the SP and BEP groups are represented in Figure 2B. A repeated-measures ANOVA performed on food intake data over 6 weeks revealed significant main effects of pretreatment (*F*_(1,44)_ = 19.57; *p* < 0.01) and week (*F*_(5,220)_ = 193.72; *p* < 0.01) but not pretreatment × week interaction. These data showed that the BEP group ate significantly more than the SP group and both groups decreased their food intake over time (*p* < 0.01 for all comparisons).

Because MC4-Rs are implicated in BW regulation (Adan et al., 2006; Krashes et al., 2016; You et al., 2016), we evaluated whether group differences in food intake were primarily associated with altered BW induced by previous binge-like ethanol exposure during adolescence. Data showing the BW of the SP and BEP groups during the intermittent-access 20% ethanol drinking paradigm are represented in Figure 2C. The repeated-measures ANOVA exhibited a significant main effect of week (*F*_(5,220)_ = 625.24; *p* < 0.01) showing that BW increased across the weeks. The pretreatment × week interaction attained statistical significance (*F*_(5,220)_ = 9.59, *p* < 0.01). The analysis of the interaction revealed that the BEP group showed reduced BW compared with the SP group during the first (*p* < 0.001) and second weeks (*p* < 0.01). No statistical significance was reported for the main factor of pretreatment. Consistent with previous reports (Maldonado-Devincci et al., 2010a), these data suggest that binge-like ethanol exposure during adolescence did not cause relevant and/or permanent disturbances in BW regulation during adulthood.

### Effects of the Selective MC4-R Agonist on Food, Ethanol and Total Calories Consumed by the Saline (SP) and Binge Ethanol-Pretreated (BEP) Groups

**Figure 3:**
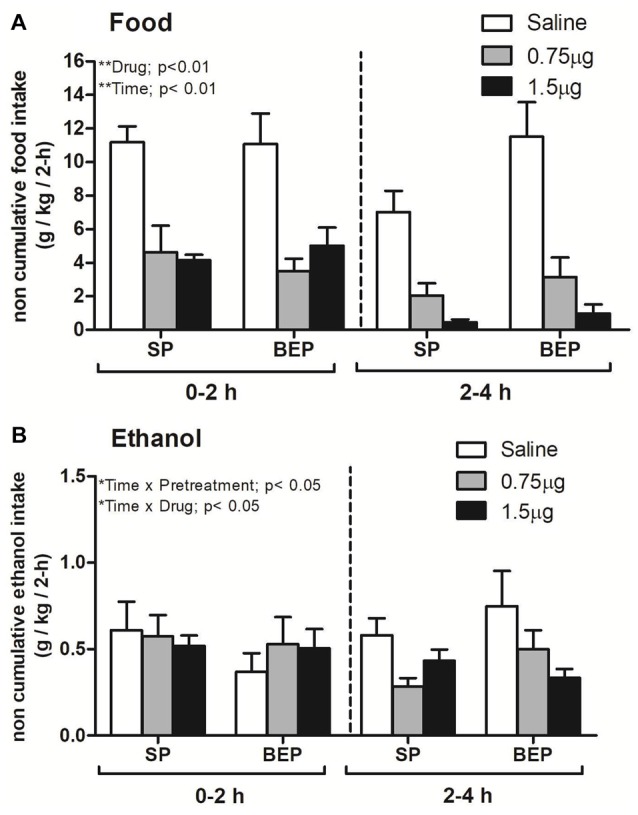
Non-cumulative food **(A)** and ethanol consumption **(B)** in the SP and BEP groups over two 2-h intervals after NAc administration of the selective melanocortin 4 receptor (MC4-R) agonist cyclo(NH-CH_2_-CH_2_-CO-His-D-Phe-Arg-Trp-Glu)-NH_2_ (0, 0.75 μg or 1.5 μg). Administration of the selective agonist significantly decreased food and ethanol consumption relative to saline administration in both the SP and BEP groups. All values are means ± SEM.

Figure 3A represents non-cumulative food consumption over two 2-h intervals following NAc infusion of the selective MC4-R agonist in the SP and BEP groups. A repeated-measures ANOVA performed on non-cumulative food consumption data revealed statistically significant main effects of drug (*F*_(2,37)_ = 48.14; *p* < 0.01) and time (*F*_(1,37)_ = 17.12, *p* < 0.01; food consumption at 2–4 h < consumption at 0–2 h). Specifically, *post hoc* Fisher’s LSD test revealed that administration of 0.75 μg and 1.5 μg significantly decreased food consumption relative to saline (*p* < 0.01) in a manner unrelated to binge-like ethanol pre-exposure.

The ANOVA performed on non-cumulative ethanol consumption data following NAc infusion of the selective MC4-R agonist (Figure 3B) showed a statistically significant drug × time interaction (*F*_(2,37)_ = 3.55; *p* < 0.05). *Post hoc* analysis revealed that both doses of the selective MC4-R agonist (0.75 μg and 1.5 μg) significantly reduced ethanol consumption relative to saline at the second 2-h interval (*p* < 0.05). A pretreatment × time interaction was also observed (*F*_(1,37)_ = 4.67; *p* < 0.05) and Fisher’s LSD test revealed that SP group decrease ethanol intake between the first and the second interval (*p* < 0.05); however, the natural tendency to decrease ethanol intake over time showed in SP group was not present in BEP group. Thus, the BEP group continues to consume large amount of ethanol both at first and second 2-h interval (*p* < 0.05).

Finally, the administration of the MC4-R agonist into the NAc was able to decrease total calories (cal/kg/4 h) consumed within 4 h after treatment (main effect of drug [*F*_(2,37)_ = 40.29; *p* < 0.01]). Concretely, both doses of the selective MC4-R agonist significantly decreased caloric intake compared with saline administration (*p* < 0.01; Table 1). Neither the main effect of pretreatment nor the pretreatment × drug interaction attained statistical significance.

**Table 1:** Total calories consumed in 4 h (cal/kg/4 h) by the SP and BEP groups after infusion of a selective MC4-R agonist (0.75 μg or 1.5 μg) or isotonic saline into the NAc. Mean ± SEM.

	BEP group	SP group
**MC4-R agonist**		
Saline	73.31 ± 7.99	60.76 ± 6.47
0.75 μg	26.47 ± 4.27^a^	25.32 ± 6.44^a^
1.5 μg	23.24 ± 4.39^a^	19.67 ± 1.04^a^

*^a^p < 0.05 with respect to saline control*.

### Blood Ethanol Concentration Following 4 h of Voluntary Ethanol Consumption in SP and BEP Animals Exposed to an Intermittent-Access 20% Ethanol Drinking Paradigm

**Figure 4:**
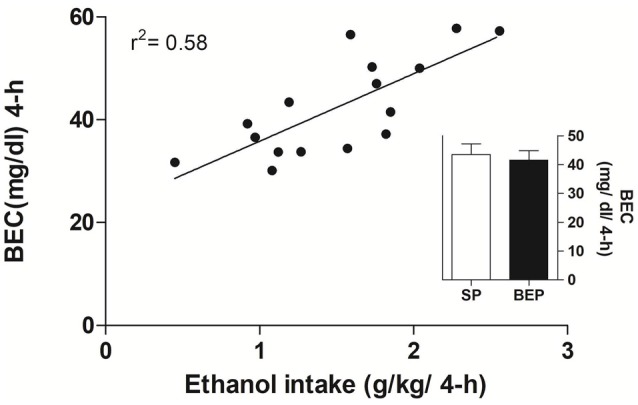
Blood ethanol concentration (BEC; mg/dl) and ethanol intake (g/kg/4 h) were evaluated across 4 h in the SP and BEP groups 1 week before NAc administration of the selective MC4-R agonist. There was no difference in BEC or ethanol consumption between groups. The amount of ethanol consumed in a 4-h drinking session significantly correlated with the measured BEC (linear regression).

Average ethanol consumed (g/kg/4 h) and BECs (mg/dl/4 h) for SP and BEP rats following 4 h of free access to ethanol using the intermittent-access 20% ethanol drinking paradigm 1 week after agonist administration are presented in Figure 4 [Ethanol intake: SP rats 1.50 ± 0.17 g/kg/4-h and BEP rats 1.52 ± 0.22 g/kg/4-h; BECs: SP rats 43.5 ± 3.66 (mg/dl/4-h) and BEP rats 41.5 ± 3.23, (mg/dl/4-h)]. Independent one-way ANOVAs performed on ethanol consumption and BEC data revealed no significant main effect of pretreatment (*F*_(1,14)_ = 0.00; *p* > 0.05 and *F*_(1,14)_ = 0.15; *p* > 0.50, respectively).

## Discussion

The most important observations in the study are as follows: (1) intermittent 20% ethanol drinking triggered high spontaneous ethanol intake in adult animals of both the SP and BEP groups; suggesting that under the experimental conditions we employed, binge-like ethanol pre-exposure during adolescence did not enhance vulnerability to ethanol consumption in adulthood; (2) a combination of binge-like ethanol pretreatment during adolescence (BEP) and high levels of ethanol intake due to intermittent ethanol exposure during adulthood triggered increased food intake without a parallel increase in BW in BEP animals; and (3) NAc infusion of a selective MC4-R agonist significantly reduced feeding, ethanol intake and total calories in adult animals with high rates of ethanol consumption, independent of pretreatment.

Regarding the first observation, several studies have previously demonstrated that standard adult laboratory rats, despite having no genetic vulnerability to high rates of spontaneous ethanol consumption, would consume high and pharmacologically relevant levels of ethanol when given intermittent access to 20% ethanol in a 2-bottle-choice drinking paradigm (Simms et al., 2008; Hwa et al., 2011; Li et al., 2011). Consistent with this, we report here that intermittent exposure to 20% ethanol induced high rates of ethanol intake in adult Sprague-Dawley rats, correlated with pharmacologically relevant BEC, as measured during the first 4 h of ethanol access.

More importantly, this behavioral profile was unrelated to any previous history of binge-like ethanol exposure during adolescence. This fact contrasts with previous research showing that binge-like ethanol exposure during adolescence increases voluntary ethanol consumption in adulthood (Pascual et al., 2009; Maldonado-Devincci et al., 2010a). It has been demonstrated that ethanol exposure during adolescence elicits neurobiological changes, such as up-regulation of neuroinflammatory mediators (Pascual et al., 2007), down-regulation of dopamine and glutamate receptors (Pascual et al., 2009) or increased expression of corticotrophin-releasing hormone (Przybycien-Szymanska et al., 2011). Moreover, we previously showed that binge-like ethanol exposure during adolescence reduces basal α-MSH and alters AgRP expression in hypothalamic and limbic areas in adult rats (Lerma-Cabrera et al., 2013a). Additionally, pharmacological studies showed that administration of a selective MC4-R antagonist into the NAc increases ethanol drinking in Sprague-Dawley rats (Lerma-Cabrera et al., 2012), suggesting that endogenous α-MSH might have a protective role against excessive ethanol intake by negatively modulating the rewarding properties of ethanol via MC4-R signaling in the NAc. When the evidence is taken together, it is tempting to propose that a long-lasting reduction of α-MSH in hypothalamic and limbic areas, triggered by binge-like ethanol pretreatment during adolescence, might enhance vulnerability to ethanol consumption during adulthood. Surprisingly, in our study, binge-ethanol administration during adolescence did not enhance ethanol consumption as tested through an intermittent 20% ethanol drinking paradigm during adulthood. Because this paradigm triggers high rates of voluntary ethanol consumption in all groups (Simms et al., 2008), the possibility that a ceiling effect obscured detection of changes in ethanol consumption between BEP and SP group exists. On the other hand, there is alternative experimental evidence that exposure to ethanol during adolescence does not necessarily increase the risk of ethanol abuse and dependence during adulthood (Siegmund et al., 2005; Clark et al., 2008; Gurkovskaya et al., 2009) and that differences do not emerge until subjects experience a stressor such as exposure to foot shock or forced swim (Siegmund et al., 2005). Furthermore Gilpin et al. (2012) reported that forced exposure to binge-like alcohol during adolescence causes long-lasting reduction, rather than increase, in voluntary ethanol consumption in adulthood (Gilpin et al., 2012). The contrasting results in these studies may be related to the use of different protocol of ethanol exposure during adolescence as well as the paradigm used to evaluate ethanol intake in adulthood. Therefore, the present data cannot rule out that our protocol of binge-like ethanol exposure during adolescence might have elicited unobserved or silent adaptations in neural mechanisms involved in ethanol consumption. But these neurobiological alterations could be only evident under certain condition such as stress or ethanol withdrawal as proposed elsewhere (Siegmund et al., 2005; Gilpin et al., 2012). Our results emphasize the need to further characterize the conditions under which binge-like ethanol exposure during adolescence might affect ethanol consumption during adulthood.

Regarding the second observation, we show here that a combination of binge-like ethanol pretreatment during adolescence (BEP) and high levels of ethanol intake due to ethanol intermittent exposure during adulthood triggered increased food intake without a parallel increase in BW in BEP animals. The present data are in accordance with clinical observations showing a high rate of co-morbidity between alcohol abuse and eating disorders (Gregorowski et al., 2013; Fouladi et al., 2015; Rush et al., 2016). A recent study determined that the rate of co-occurrence between eating disorders and binge drinking in young people between 18 and 22 years old is as high as 18% (Rush et al., 2016). Additionally, it has been demonstrated that severity and frequency of alcohol consumption in adolescents are positively associated with number of eating disorder symptoms (Arias et al., 2009). Although these epidemiological studies show the relationship between alcohol intake during adolescence and eating disorders, studies evaluating the impact of alcohol consumption during adolescence on eating disorders in adulthood are scarce. Previous studies carried out in animal models had shown the existence of a set threshold for the stimulation of appetite and food intake by alcohol (Caton et al., 2004). This could explain why adult rats that consume high levels of ethanol and were pre-exposed to binge ethanol during adolescence are more sensitive to the stimulatory effect of alcohol on food intake than those not pre-exposed to ethanol during adolescence.

Further experiments are needed to evaluate the potential implication of ethanol exposure during adolescence regarding energy balance in adulthood. However, given that enhanced feeding in the BEP group was not parallel to increased BW, there is the possibility that binge-like ethanol exposure during adolescence might have elicited brain alterations, such as changes in hypothalamic neuropeptides function, triggering caloric efficiency disturbances in adult rats exhibiting high ethanol consumption. One possible explanation for why BEP group exhibited increased food intake but no difference in BW with respect to SP would be group differences in spontaneous locomotor activity. However, ethanol exposure during adolescence reduces, rather than increases, spontaneous locomotor activity (Pascual et al., 2009; Teixeira et al., 2014), suggesting that other factors may be responsible for our data. Another hypothesis that could explain the present data relies on the effect of binge-like ethanol exposure during adolescence on the MC system, a key neural pathway involved in the regulation of food intake and energy balance (De Jonghe et al., 2011; Krashes et al., 2016). First, binge-like ethanol exposure during adolescence significantly reduces basal α-MSH in hypothalamic and limbic areas and increases AgRP immunoreactivity in response to a high dose of ethanol during adulthood (Lerma-Cabrera et al., 2013a). Second, i.c.v. administration of α-MSH in rats (Abbott et al., 2000) or of MTII, a nonselective MCR agonist, in mice reduces food intake (Navarro et al., 2003). Moreover, MC4-R knockout mice do not reduce their food intake following i.c.v. administration of MTII (Marsh et al., 1999; Navarro et al., 2011). Conversely, increased food intake has been reported after administration of a selective antagonist of MC4-R into the lateral hypothalamus, NAc and VTA (Lerma-Cabrera et al., 2012). Finally, it has recently been demonstrated that AgRP cell activity is essential for ethanol-induced overeating (Cains et al., 2017). Together, these data suggest the critical role of MC4-R signaling in energy balance. Given this fact and our present data, there is a possibility that binge-like ethanol exposure during adolescence combined with high rates of ethanol intake during adulthood alters MC function, which might contribute to energy metabolism disturbances during adulthood.

Finally, we report here that NAc infusion of a selective MC4-R agonist triggered a strong anorectic response and reduced total calories consumed over a 4-h testing period in animals showing high rates of ethanol consumption (more than 4 g/kg/24 h), unrelated to previous history of binge-like ethanol exposure during adolescence. It has been reported that administration of an MC4-R agonist does not cause significant changes in locomotor activity (Klenerová et al., 2008; Bertolini et al., 2009); therefore, it is unlikely that reduced food and ethanol intake following MC4-R activation were the result of generalized behavioral inhibition. Our data, showing reduced ethanol and food intake in high ethanol drinkers as a result of the administration of a selective MC4-R agonist, extend our previous observation reported in low ethanol drinkers (Lerma-Cabrera et al., 2012). Additionally, they are consistent with our previous studies demonstrating that an MC4-R agonist blunts ethanol drinking in high-ethanol-consuming C57BL/6J mice (Navarro et al., 2005) and that MTII, a nonselective MC4/3-R agonist, fails to reduce ethanol drinking in MC4-R knockout mice (Navarro et al., 2011). The present findings add relevant preclinical information regarding the relevance of MC4-R signaling into the NAc as a potential target for therapeutic interventions for eating disorders (Adan et al., 2006; Girardet and Butler, 2014) and alcohol use disorders (AUDs) in humans (Olney et al., 2014a).

Importantly, we have previously demonstrated that binge-like ethanol exposure during adolescence reduces basal α-MSH and alters AgRP expression in hypothalamic and limbic areas in adult rats (Lerma-Cabrera et al., 2013a). However, pre-exposure to ethanol during adolescence did not alter the effect of administration of selective MC4-R agonist into NAc on feeding and ethanol consumption. It is possible that reduction in basal α-MSH-induced by binge-like ethanol pre-exposure during adolescence was not sufficient to blunt the effect of selective agonist of MC4-R into NAc. Additional studies aimed to quantify protein level of α-MSH and MC4-R after NAc administration of selective agonist of MC4-R in adult rats pre-exposed to ethanol during adolescence may help to test this hypothesis. Another possible explanation for the present data relies on the fact that α-MSH exerts its effects by binding to both MC3-R and MC4-R. However, we used a selective agonist of MC4-R leaving MC3-R functioning unimpeded. It is known that MC3-R regulate the endogenous α-MSH signaling onto MC4R, probably acting as an inhibitory autoreceptor on POMC neurons (Renquist et al., 2011). Moreover, a recent study demonstrated that MC3R−/− mice were more sensitive to the protective effects of MTII than MC3R+/+ mice (Olney et al., 2014b) suggesting that MC3-R may contribute to binge-like ethanol drinking. Given that in this study we used an intermittent ethanol access schedule, a model of binge-like alcohol drinking in rats (Simms et al., 2014), the contribution of MC3-R to ethanol consumption in adults rats pre-exposed to binge-like ethanol paradigm during adolescence should be evaluated in future studies.

Because ethanol has both caloric and reward properties, the mechanisms underlying the effect of NAc administration of a selective MC4-R agonist on ethanol intake remain unclear. Taking into account that direct ingestive responses are not valid direct measures of the hedonic value of ethanol/food (Salamone et al., 2016), we cannot conclude whether intake disturbances observed after NAc infusion of the selective MC4-R agonist were calorically driven or whether they were the result of hedonically driven non-homeostatic processes. Furthermore, given our previous data (Lerma-Cabrera et al., 2013b), it is tempting to speculate that MC4-R signaling within the NAc shell has a key role in non-homeostatic aspects (palatability) of ethanol as well as food intake also in high ethanol drinkers. Although the precise nature of the neurochemical mechanisms involved remains uncertain, MC/opioid interactions within the NAc have been proposed and discussed elsewhere (Lerma-Cabrera et al., 2012). Anatomical (Grossman et al., 2003; Bertolini et al., 2009) and pharmacological studies (Ploj et al., 2002; Starowicz et al., 2003; Kalange et al., 2007) have suggested the existence of an important neurobiological interaction between MC and opioids. Recently, it has been shown that administration of MTII synergistically augments the ability of naltrexone to blunt binge-like ethanol intake in mice (Navarro et al., 2015). Given the role of opioids in ethanol palatability (Peciña, 2008; Katsuura and Taha, 2014; Ikeda et al., 2015; Uhari-Väänänen et al., 2016), one interesting avenue for future research will be to explore the contribution of MC-opioid interactions within the NAc to the hedonic aspects of ethanol consumption in high ethanol drinkers.

We report here that a combined history of binge-like ethanol pre-exposure during adolescence and high ethanol consumption during adulthood elicits an increase in food intake without BW changes in adulthood, which suggests caloric efficiency disturbances. Additionally, this study reports that MC4-R stimulation within the NAc reduces feeding and ethanol intake in high ethanol-drinking adult rats, unrelated to previous episodes of binge-like ethanol exposure during adolescence, which adds new evidence regarding the dual ability of MC compounds to control excessive ethanol and food intake.

## Author Contributions

IC, MN and TET made substantial contribution to the study concept and design. FC, JML-C and MA-I conducted the experiments. FC, JML-C, MA-I and IC analyzed the data. All the authors critically reviewed content and approved the final version for publication.

## Conflict of Interest Statement

The authors declare that the research was conducted in the absence of any commercial or financial relationships that could be construed as a potential conflict of interest.
